# Synergistic Enhancement of Corrosion Resistance of GO/LDH Coating on Anodized Magnesium Alloy Surfaces via pH-Regulated In Situ Growth and Anionic Corrosion Inhibitor Intercalation

**DOI:** 10.3390/ma19122525

**Published:** 2026-06-11

**Authors:** Yanning Chen, Tongqing Wang, Manyu Liu, Hao Ji, Yuehua Sun, Zhen Sun, Chengsi Zheng, Zhenya Zhang, Mingya Zhang

**Affiliations:** 1 Key Laboratory of Green Fabrication and Surface Technology of Advanced Metal Materials, Ministry of Education, School of Materials Science and Engineering, Anhui University of Technology, Maanshan 243002, China; 2 Inner Mongolia Key Laboratory of New Materials and Surface Engineering, School of Materials Science and Engineering, Mongolia University of Technology, Hohhot 010051, China

**Keywords:** magnesium alloy, layered double hydroxides, graphene oxide, corrosion inhibitor, corrosion resistance

## Abstract

Magnesium alloys offer low density, high strength, excellent heat dissipation, and good electrical conductivity, benefiting automotive and aerospace sectors. However, magnesium and its alloys are highly susceptible to corrosion, which severely limits their practical use. In this study, the hydrothermal deposition of graphene oxide (GO) and layered double hydroxides (LDHs) was achieved on the surface of an anodized magnesium alloy, forming a GO/LDH coating. The effects of pH and various anionic corrosion inhibitors on the corrosion resistance of the GO/LDH coating were subsequently investigated. The results show that the GO/LDH coating prepared at pH 10.8 exhibits the best corrosion resistance, which is generally associated with a greater coating thickness, with its nanosheets growing in a wavy manner in all directions. This coating also shows higher crystal transparency and a denser layered structure. Based on this, anionic corrosion inhibitors including molybdate, vanadate, and tungstate were incorporated into the GO/LDH coating. Electrochemical impedance (EIS) analysis subsequently revealed that the GO/LDH–molybdate coating exhibited the highest |Z|_0.01 HZ_, reaching ~10^5.5^ Ω cm^2^, indicating its excellent corrosion resistance. This approach offers a novel and effective route to significantly improve the corrosion resistance of magnesium alloys via synergistic coating design.

## 1. Introduction

Magnesium alloys have attracted significant attention in the aerospace and automotive industries due to their low density, high specific strength, good thermal conductivity, and excellent electromagnetic shielding properties [1,2]. As the most widely used commercial wrought magnesium alloy, AZ31 magnesium alloy has the advantages of excellent formability, moderate strength, low cost and good biocompatibility, and has become the core candidate material for lightweight structural parts and biodegradable implants. However, its high chemical reactivity and poor corrosion resistance in chloride-containing service environments lead to premature failure of components, which has become the key bottleneck restricting its large-scale engineering application. In corrosive environments, magnesium alloys readily form galvanic cells with alloying elements or impurities, leading to rapid localized corrosion [3,4]. Moreover, the corrosion products of magnesium alloys are typically loose and porous, lacking effective protective properties and thus failing to prevent further corrosion [5].

To address this challenge, extensive research has been devoted to enhancing the corrosion resistance of magnesium alloys. Various surface protection strategies have been developed, including chemical conversion coatings, anodizing, metal plating, and organic/polymer coatings [6,7]. Among these, the application of corrosion-resistant coatings is considered the most effective approach, as it can physically isolate the alloy substrate from corrosive media. However, conventional single-layer coatings often suffer from inherent porosity, inadequate adhesion, or insufficient long-term durability, which limits their protective performance.

In recent years, LDHs have emerged as promising materials for corrosion protection. LDHs are a class of anionic clays with a unique layered structure consisting of positively charged hydroxide layers and exchangeable interlayer anions [8,9]. They exhibit several advantageous properties, including high chemical stability, excellent anion exchange capacity, self-healing ability, and the capability to form dense, well-adhered films on metal substrates via substrate-assisted hydrothermal deposition [10,11]. When grown on magnesium alloys, LDH coatings can effectively seal micro-pores and defects, providing both a physical barrier and active corrosion protection through the release of intercalated inhibitors [12,13].

GO, a derivative of graphene, has also attracted considerable interest in the field of corrosion protection. GO possesses a large specific surface area, high mechanical strength, and abundant oxygen-containing functional groups (such as –COOH, –OH, and C–O–C) on its surface and edges [14,15]. These functional groups enable strong interactions with metal substrates and other coating materials through hydrogen bonding, coordination bonding, or covalent bonding. Additionally, GO can serve as an excellent carrier material, promoting uniform dispersion of LDH nanosheets and enhancing the compactness of composite coatings via its 2D lamellar barrier effect [15,16].

The combination of LDHs and GO into composite coatings has been proven to be a promising strategy to synergistically enhance corrosion resistance of metal substrates [17,18,19]. In such composite structures, the LDH layer provides a stable in situ growth base with anion exchange and self-healing functionality, while the GO nanosheets act as physical barriers to prolong the diffusion path of corrosive ions, and simultaneously serve as nucleation sites to regulate the growth of LDH crystals. However, current related studies still have three key limitations that need to be addressed: (1) the formation mechanism of GO/LDH composite coating is significantly affected by the pH of the reaction solution, but most studies only focus on the effect of pH on the coating morphology, lacking systematic analysis of pH on the phase composition, crystallinity and interfacial bonding of the composite coating; (2) the synergistic protection mechanism of pH-regulated structure optimization and anionic inhibitor intercalation has not been fully elucidated, and the structure–activity relationship between coating microstructure and long-term corrosion resistance remains unclear; (3) there is still a lack of systematic comparative studies on the modification effect of different anionic inhibitors on GO/LDH composite coatings for magnesium alloy protection [17,18,20].

To further enhance the protective performance, anionic corrosion inhibitors can be intercalated into the interlayer spaces of LDHs. When incorporated, these inhibitors can be released in response to corrosive stimuli, providing active protection in addition to the passive barrier effect [16,17]. Common anionic inhibitors such as molybdate, vanadate, and tungstate have been reported to effectively suppress corrosion processes by forming stable passive films or inhibiting cathodic reactions [18]. Nevertheless, systematic studies on the combined effects of pH-controlled substrate-assisted hydrothermal deposition and inhibitor intercalation on the structural evolution and corrosion resistance of GO/LDH coating remain limited [21].

In this study, GO/LDH composite coatings were grown in situ on anodized AZ31 magnesium alloy surfaces. The effects of pH (9.8, 10.8, and 11.8) on the microstructure and corrosion resistance of the coatings were systematically investigated. Based on the optimized pH, three anionic corrosion inhibitors, molybdate, vanadate, and tungstate, were intercalated into the GO/LDH interlayers. This work aims to establish a synergistic protection mechanism combining a dense physical barrier with active inhibition, offering a new approach for developing high-performance anti-corrosion coatings for magnesium alloys.

## 2. Materials and Methods

### 2.1. Preparation of Coating

AZ31 magnesium alloy with a nominal chemical composition (wt.%) of Al 2.5–3.5, Zn 0.6–1.4, Mn ≥ 0.2, Si ≤ 0.1, Cu ≤ 0.05, Ni ≤ 0.005, Fe ≤ 0.005, and Mg balance was used as the substrate material. The alloy was received in the hot-rolled state and cut into specimens with dimensions of 20 mm × 20 mm × 5 mm and 10 mm × 10 mm × 5 mm for subsequent experiments. Prior to coating deposition, the specimens were sequentially ground with SiC sandpapers from 400# to 2000# grit. After polishing, the sample was cleaned with anhydrous ethanol and then dried with cold air for later use. Then, 7.14 g of sodium hydroxide (Sinopharm Chemical Reagent Co., Ltd., Shanghai, China) was added to one liter of ultrapure water, and 4 g of sodium aluminate (Sinopharm Chemical Reagent Co., Ltd., Shanghai, China) was added to one liter of ultrapure water and stirred. The rotor was placed in a constant temperature magnetic stirrer, and the stirred solvent was added with a rotation speed of about 3.5. The anode was connected to the polished AZ31B specimens, and the cathode was connected to a copper sheet. A DC voltage of 20.0 V was applied for 30 min to perform anodic oxidation treatment. The treated sample was cleaned with anhydrous ethanol and then dried with cold air, providing active sites for the subsequent in situ growth of LDHs.

**Scheme 1:**
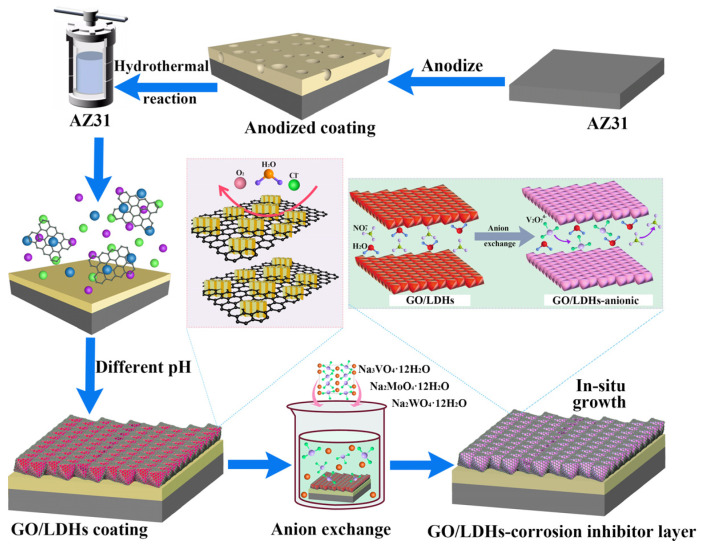
Schematic illustration of the fabrication process of the GO/LDH composite coating and subsequent intercalation of anionic corrosion inhibitors.

A 0.05 M aluminum nitrate (Sinopharm Chemical Reagent Co., Ltd., Shanghai, China) solution and a 0.3 M sodium nitrate (Sinopharm Chemical Reagent Co., Ltd., Shanghai, China) solution were prepared and thoroughly mixed. Subsequently, the pH of the mixture was precisely adjusted to 10.8 by adding a 0.3 M sodium hydroxide solution. This solution was loaded into an autoclave together with the polished magnesium alloy samples, and a hydrothermal reaction was conducted at 125 °C for 12 h in a high-temperature oven. After the reaction was completed, the resulting coating was first subjected to ultrasonic cleaning for 5 min to remove any potential surface residues, then alternately rinsed three times with deionized water and ethanol and finally dried under a stream of cold air to obtain the LDH coating (Scheme 1).

A 0.1 mg/mL GO solution was prepared by appropriately diluting a 7 mg/mL GO solution that had been stored in a refrigerator. Meanwhile, a mixed solution of 0.05 M aluminum nitrate and 0.3 M sodium nitrate was prepared. Subsequently, the pH of the mixed solution was adjusted using a 0.3 M sodium hydroxide solution to achieve values of 9.8, 10.8, and 11.8, respectively. These solutions were placed in a hydrothermal synthesis reactor together with finely polished magnesium alloy samples and subjected to hydrothermal treatment in an oven at 125 °C for 12 h. After the reaction was completed, the resulting coatings were removed. They were first ultrasonically cleaned for 5 min to remove surface impurities, then alternately washed three times with deionized water and ethanol and finally dried under a stream of cold air to obtain GO/LDH coating grown under different pH conditions (pH 9.8 GO/LDHs, pH 10.8 GO/LDHs, pH 11.8 GO/LDHs).

The optimal GO/LDH coating obtained under the optimal pH was selected. A 0.1 M Na_3_VO_4_·12H_2_O solution was prepared, and its pH was adjusted to 10 by dropwise addition of 3 M NaOH solution. The GO/LDH coating was placed in the Na_3_VO_4_·12H_2_O solution and subjected to hydrothermal treatment in a hydrothermal reactor (60 °C, 6 h), denoted as the GO/LDH–vanadate coating. A 0.1 M Na_2_MoO_4_·12H_2_O solution was prepared, and its pH was adjusted to 10 by dropwise addition of 3 M NaOH. The GO/LDH coating was placed in the Na_2_MoO_4_·12H_2_O solution and subjected to hydrothermal treatment in a hydrothermal reactor (60 °C, 6 h), denoted as the GO/LDH–molybdate coating. A 0.1 M Na_2_WO_4_·12H_2_O solution was prepared, and its pH was adjusted to 10 by titration with 3 M NaOH. The GO/LDH coating was placed in the Na_2_WO_4_·12H_2_O solution and subjected to hydrothermal treatment in a hydrothermal reactor (60 °C, 6 h), denoted as the GO/LDH–tungstate coating. The preparation conditions for all coating samples are summarized in Table S1.

### 2.2. Surface Characterization

The surface morphology of the coating was characterized using a scanning electron microscope (SEM, JSM-6700, JSM-6700F, JEOL Ltd., Tokyo, Japan). The SEM specimens were the as-prepared coating samples, which were sprayed with gold for 60 s before testing to enhance the electrical conductivity of the coating surface. A Fourier transform infrared spectrometer (FT-IR, Shimadzu IRTracer-100, Kyoto, Japan) was used to analyze the functional groups and chemical composition of the samples. FT-IR tests were performed in the wavenumber range of 4000–500 cm^−1^ with a resolution of 4 cm^−1^ and 32 scans. The test specimens were prepared by scraping the coating from the substrate, grinding it into powder, and mixing it with KBr at a mass ratio of 1:100 to press into transparent pellets.

The crystal structure of the coating was determined using an X-ray diffractometer (XRD, Rigaku SmartLab, Akishima, Japan). XRD tests were performed with Cu Kα radiation (λ = 0.15406 nm), operating at 40 kV and 30 mA, with a scanning range of 5–90° (2θ) and a scanning speed of 5°/min. The test specimens were the as-prepared coating samples with a size of 20 mm × 20 mm × 5 mm, and the test surface was the coating surface without any additional treatment.

### 2.3. Corrosion Test

Electrochemical testing was performed using an electrochemical workstation (CorrTest CS310M, Wuhan CorrTest Instruments Corp., Ltd., Wuhan, China) equipped with a standard three-electrode cell system. The electrolyte used was a 3.5 wt% aqueous NaCl solution, which is widely used to simulate the corrosive marine service environment and chloride-containing physiological environment of magnesium alloy components, and can effectively evaluate the anti-corrosion performance of the coating in practical service scenarios. A saturated calomel electrode (SCE) was used as the reference electrode, a platinum plate (10 mm × 10 mm) was used as the counter electrode, and the coated magnesium alloy specimen was used as the working electrode. Prior to electrochemical testing, the back and sides of the specimen were sealed with epoxy resin, leaving an exposed working area of 1 cm^2^ on the coating surface. The sealed specimens were immersed in the 3.5 wt% NaCl solution for 30 min to stabilize the open circuit potential (OCP) before the test.

Electrochemical impedance spectroscopy (*EIS*) testing was performed over a frequency range of 100 kHz to 10 mHz with a sinusoidal excitation amplitude of 10 mV. Dynamic potential polarization testing was conducted at a scan rate of 1 mV/s within the potential range of −0.2 V to +0.2 V (relative to the SCE). All potentiodynamic polarization measurements were conducted with automatic iR-drop compensation using the current-interrupt method integrated into the CorrTest CS310M electrochemical workstation. Compensation was verified before each measurement, with residual solution resistance confirmed to be less than 5 Ω. The slight instability observed in the anodic branches of certain Tafel curves is attributed to localized pitting initiation and/or hydrogen bubble accumulation on the coating surface. Critically, the linear Tafel region (approximately ±60–120 mV from *E_corr_*) used for kinetic parameter extraction remained unaffected by this instability, ensuring the validity of the calculated *i_corr_* and *E_corr_* values. The EIS test recorded a sinusoidal signal with a frequency ranging from 100 kHz to 0.01 Hz and an amplitude of 10 mV, yielding the final impedance data. Finally, the Tafel extrapolation method was used to calculate the corrosion current density (*i_corr_*) and corrosion potential (*E_corr_*) from the polarization curves. The measured data were fitted to an equivalent circuit using CView software 3.3c to interpret the corrosion characteristics of the coating in a 3.5 wt% aqueous NaCl solution. All electrochemical measurements were repeated at least three times using independently prepared specimens. Results are reported as mean ± standard deviation (SD).

## 3. Results

### 3.1. Synthesis and Characterization

**Figure 1:**
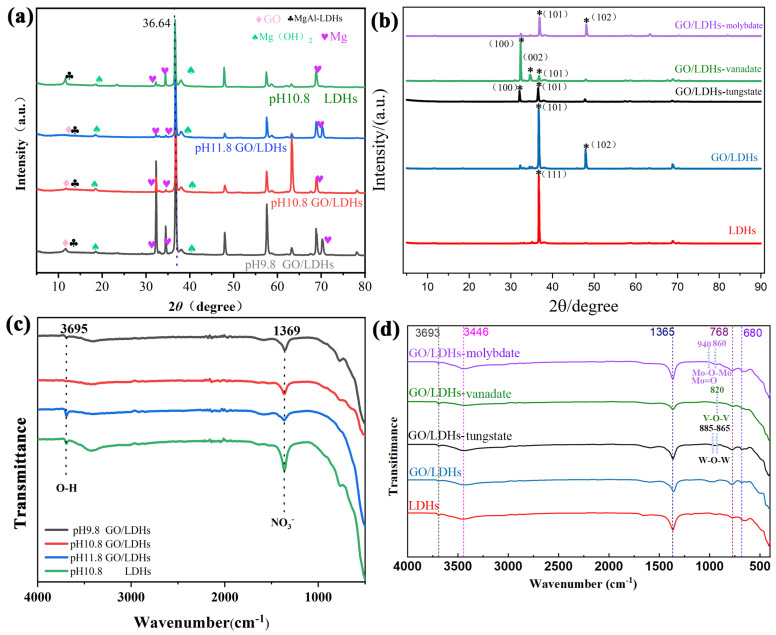
(**a**) XRD patterns of coatings grown under different pH conditions; (**b**) XRD patterns of LDHs, GO/LDHs, GO/LDH–tungstate layers, GO/LDH–vanadate layers, and GO/LDH–molybdate layers (“*” indicates the position of diffraction peaks); (**c**) FT-IR spectra of coatings grown under different pH conditions; (**d**) FT-IR spectra of the coatings intercalated with different corrosion inhibitors.

The XRD patterns of the coatings obtained under different pH conditions are shown in Figure 1. Crystalline phases of MgAl-LDHs, Mg(OH)_2_, and Mg were identified in the LDH coating, with a diffraction peak corresponding to Mg(OH)_2_ observed at approximately 36.64° in the 2θ angle. In addition, two distinct diffraction peaks appeared at 11.6° and 33.3°, corresponding to the (003) and (006) crystal planes of LDHs [22], respectively, confirming the formation of the MgAl-LDH phase on the coating surface. All GO/LDH coatings exhibited a clear diffraction peak at 10.5°, corresponding to the (001) crystal plane of GO [20,23]. Furthermore, diffraction peaks corresponding to the (003) and (006) crystal planes of LDHs were observed in all composite coatings, indicating the successful formation of GO/LDH nanocomposite structures on the magnesium alloy substrate.

With the increase in pH from 9.8 to 10.8, the characteristic diffraction peaks of the LDH (003) and (006) crystal planes become sharper and stronger, and the relative crystallinity of MgAl-LDHs increases significantly, indicating that pH 10.8 is more conducive to the ordered growth of LDH crystals. Meanwhile, the characteristic peak of the GO (001) crystal plane is the most obvious at pH 10.8, which proves that GO can be effectively retained and compounded with LDHs under this pH condition. When the pH further increases to 11.8, the intensity of LDH characteristic diffraction peaks decreases significantly, and the half-peak width increases, indicating that the crystallinity of LDHs decreases and the crystal growth is disordered. In addition, the diffraction peak intensity of Mg(OH)_2_ increases significantly at pH 11.8, which is attributed to the excessive OH^−^ in the solution reacting with the magnesium alloy substrate to form a large amount of Mg(OH)_2_ by-product, which destroys the ordered growth of LDHs and the GO composite structure. This result confirms that pH has a significant regulatory effect on the phase composition and crystallinity of the GO/LDH composite coating.

Figure 1b presents the XRD patterns of the composite coatings intercalated with different corrosion inhibitors. The results show that the LDH coating exhibits a distinct diffraction peak at 37.1°, corresponding to the (101) crystal plane of GO/LDHs, which represents the interlayer phase of LDHs. For the GO/LDH coatings intercalated with molybdate, vanadate, and tungstate, the characteristic diffraction peak of the (101) crystal plane was also observed, although the peak intensities varied, reflecting the influence of different anionic intercalation on crystal orientation or crystallinity [19]. In addition, the (100) and (102) diffraction peaks of LDHs are present in all composite coatings, confirming the successful formation of GO/LDHs and the intercalated nanosheets with different corrosion inhibitors on the magnesium alloy surface.

Figure 1c presents the FT-IR spectra of GO/LDH films prepared under different pH conditions. All coatings exhibit distinct absorption peaks at approximately 3695 cm^−1^ and 1369 cm^−1^, corresponding to the stretching vibrations of O–H bonds and NO_3_^−^, respectively. These peaks are primarily attributed to the presence of adsorbed water on the coating surface or interlayer water molecules, and also reflect the stretching vibrations of Mg–O and Al–O in LDHs. The results indicate the coexistence of GO and LDH phases on the coating surface, confirming that doping of LDHs by GO was achieved [24]. Furthermore, the coating prepared at pH 10.8 shows significantly higher peak intensities associated with oxygen-containing functional groups of GO. With the increase in pH from 9.8 to 10.8, the absorption peak intensities of oxygen-containing functional groups of GO (such as -COOH, C-O-C) at 1720 cm^−1^ and 1220 cm^−1^ are significantly enhanced, indicating that pH 10.8 is conducive to the retention of GO and its effective interfacial bonding with LDHs. When the pH rises to 11.8, the absorption peak intensity of the oxygen-containing functional groups of GO decreases significantly, which is due to the deprotonation of the oxygen-containing functional groups of GO under strong alkaline conditions, leading to its partial dissolution in the reaction solution, which is consistent with the XRD results.

Furthermore, the films prepared at pH 10.8 show significantly higher peak intensities associated with oxygen-containing functional groups of GO, suggesting that this pH condition favors the retention of GO and its effective incorporation with LDHs. This observation is consistent with the structural characteristics revealed by XRD analysis.

The Fourier transform infrared (FT-IR) spectra of the different composite coatings are shown in Figure 1d. All coatings exhibited absorption peaks at approximately 3693 cm^−1^ and 3446 cm^−1^, which are attributed to the stretching vibrations of metal hydroxyl groups in the LDH layers and the stretching vibrations of interlayer water molecules or hydroxyl groups on the GO surface, respectively. The absorption peak near 1365 cm^−1^ corresponds to the bending vibration of interlayer crystalline water, while the peaks at 768 cm^−1^ and 680 cm^−1^ are associated with the Mg-O lattice vibration in the LDH layers and the stretching vibration of interlayer anions (e.g., NO_3_^−^). The C 1s spectrum of GO/LDHs at pH 9.8 shows three main components: C–O (284.5 eV), C=O (286.7 eV), and a peak at 284.5 eV assigned to C–O with a relative intensity of 0.05. The dominant C–O and C=O contributions indicate partial oxidation of carbon species, consistent with graphene oxide presence in the coating (Figure S1). Specifically, for the GO/LDH–vanadate coating, the absorption band at 820 cm^−1^ is assigned to the asymmetric stretching vibrations of V-O-V [25]. For the GO/LDH–molybdate coating, the band at 860 cm^−1^ is attributed to the Mo-O-Mo stretching vibration and the band at 940 cm^−1^ to the Mo=O stretching mode [26]. For the GO/LDH–tungstate coating, the bands in the 865–885 cm^−1^ region are assigned to W-O-W stretching vibrations [27]. These results indicate that all three anionic corrosion inhibitors were successfully intercalated into the GO/LDH coating structure.

**Figure 2:**
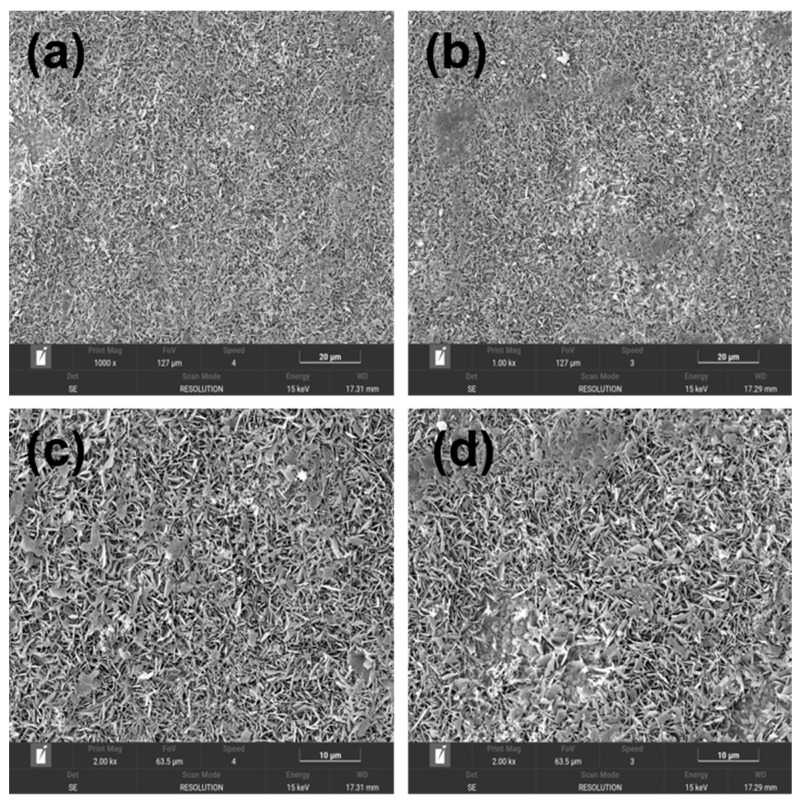
SEM images of coatings grown under different pH conditions: (**a**) pH 9.8 GO/LDHs; (**b**) pH 10.8 GO/LDHs; (**c**) pH 11.8 GO/LDHs; (**d**) pH 10.8 LDHs.

The surface morphologies of the GO/LDH composite coatings prepared on magnesium alloy surfaces under different pH conditions were characterized through scanning electron microscopy (SEM), as shown in Figure 2. The pH 9.8 GO/LDH coating exhibited a relatively sparse flake-like structure with unevenly distributed nanosheets and obvious pore defects (Figure 2a,b). When the pH was increased to 10.8, the coating surface became dense and uniform, displaying a typical wrinkled nanosheet morphology (Figure 2c,d). The nanosheets grew in multiple directions on the substrate, forming a tightly interwoven structure, and the coating thickness increased significantly. As the pH was further raised to 11.8, local agglomeration appeared on the coating surface, accompanied by an increase in nanosheet size but disordered arrangement, and microcracks were observed in some areas (Figure 2c). In contrast although the pure LDH coating also exhibited a flake-like structure with nanosheets growing perpendicular to the substrate [8,24], its density was inferior to that of the GO/LDH composite coating prepared at pH 10.8 (Figure 2b). These results indicate that pH has a significant influence on the microstructure of the GO/LDH composite coating, and that pH 10.8 represents the optimal growth condition, favoring the formation of a dense, intact composite coating with fewer defects [28].

The significant effect of pH on the coating microstructure is mainly attributed to the dual regulation of pH on the nucleation and growth kinetics of LDH crystals and the interfacial interaction between GO and LDHs. (1) Regulation of LDH crystallization kinetics: The formation of MgAl-LDHs follows the reaction equation 6Mg^2+^ + 2Al^3+^ + 16OH^−^ + 2NO_3_^−^ + 4H_2_O → Mg_6_Al_2_(OH)_16_(NO_3_)_2_·4H_2_O. At pH 9.8, the concentration of OH^−^ in the solution is low, resulting in a low supersaturation of the LDH precursor, slow nucleation rate, and uneven growth of nanosheets, thus forming a sparse structure with obvious pore defects. At pH 10.8, the OH^−^ concentration matches the hydrolysis rate of metal ions, and the nucleation rate and growth rate of LDH crystals reach an optimal balance, which is conducive to the formation of uniform and ordered LDH nanosheets; meanwhile, the weak alkaline environment avoids the excessive corrosion of the magnesium substrate, ensuring the stable growth of the coating. At pH 11.8, the excessive OH^−^ leads to ultra-high supersaturation of the precursor, rapid nucleation of LDH crystals, and disordered growth of nanosheets, accompanied by severe corrosion of the substrate and the formation of a large amount of Mg(OH)_2_ by-product, resulting in agglomeration and microcracks in the coating. (2) Regulation of GO-LDH interfacial interaction: The surface of GO nanosheets is rich in oxygen-containing functional groups, which are negatively charged after deprotonation in alkaline solution and can be used as nucleation sites for positively charged LDH laminates through electrostatic adsorption. At pH 10.8, the deprotonation degree of GO oxygen-containing functional groups is moderate, which not only ensures good dispersion of GO in the solution but also provides sufficient active sites for the ordered growth of LDHs, promoting the formation of a tightly interwoven composite structure. At pH 9.8, the insufficient deprotonation of GO leads to weak electrostatic interaction with LDHs, and the nanosheets are unevenly distributed. At pH 11.8, the excessive deprotonation of GO leads to its partial dissolution in the strong alkaline solution, which reduces the regulatory effect on LDH growth, resulting in disordered arrangement of nanosheets.

**Figure 3:**
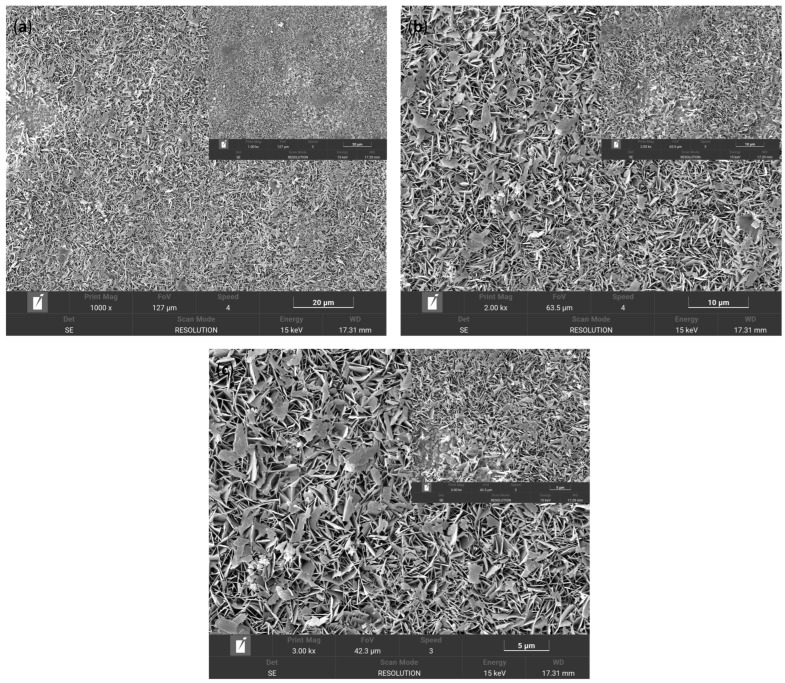
SEM images of the samples: (**a**) GO/LDH–vanadate layers; (**b**) GO/LDH–molybdate layers; (**c**) GO/LDH–tungstate layers.

SEM images of GO/LDH coating intercalated with different anionic corrosion inhibitors are presented in Figure 3. In Figure 3a, it is observed that the GO/LDH–vanadate layers showed a noticeable change in surface morphology, with slightly enlarged nanosheets arranged more compactly and fewer surface defects. As displayed in Figure 3b, the GO/LDH–molybdate layers demonstrated the most uniform and dense surface morphology, characterized by hexagonal nanosheets tightly stacked together, with almost no observable pores or cracks, indicating optimal structural integrity. In contrast, although the GO/LDH–tungstate layers in Figure 3c also formed a relatively dense lamellar structure, the arrangement of the nanosheets was slightly less orderly than that of the GO/LDH–molybdate layers, with minor undulations observed locally. These morphological characteristics indicate that the intercalation of different anionic corrosion inhibitors significantly influenced the microstructure of the GO/LDH coating, among which the GO/LDH–molybdate layers favored the formation of the most compact and defect-free coating structure, which was closely associated with its excellent corrosion resistance.

### 3.2. Electrochemical Corrosion Test

**Figure 4:**
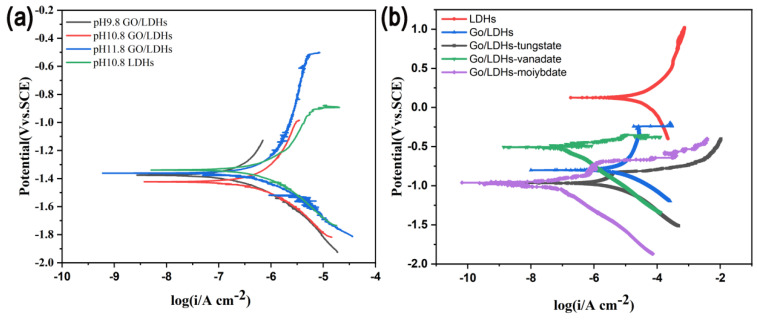
(**a**) Polarization curves of film layers grown under different pH conditions after soaking in a 3.5 wt.% NaCl solution for 30 min; (**b**) polarization curves of different film layers after soaking in a 3.5 wt.% NaCl solution for 30 min.

Based on the potentiodynamic polarization curves shown in Figure 4a,b and Figure S2, combined with the corrosion current density (*i_corr_*) and corrosion potential (*E_corr_*) calculated using the Tafel extrapolation method, the effects of different pH conditions and various anionic corrosion inhibitors on the corrosion resistance of the GO/LDH coating can be analyzed [29,30]. According to the literature, *E_corr_* is mainly influenced by the thermodynamic properties of the coating material; therefore, the corrosion potential was not used to evaluate the corrosion resistance of the coatings in this study [31,32,33]. Generally, the lower the *i_corr_*, the better the corrosion resistance. The GO/LDH coating prepared at pH 10.8 exhibits the lowest corrosion current density of 3.5197 × 10^−7^ A/cm^2^ (as shown in Table 1 and Table S2), which is significantly lower than that of the GO/LDH coating prepared at pH 9.8 (6.0338 × 10^−7^ A/cm^2^) and that prepared at pH 11.8 (5.8592 × 10^−7^ A/cm^2^). This indicates that the coating fabricated at pH 10.8 possesses the optimal corrosion resistance [34,35]. The non-monotonic dependence of *i_corr_* on pH can be rationalized through the thermodynamic stability and crystallization kinetics of MgAl-LDHs. At pH 9.8, the supersaturation relative to MgAl-LDHs is relatively low, resulting in insufficient nucleation density and the formation of a loosely packed film with abundant through-thickness defects. At pH 11.8, the precipitation kinetics become excessively rapid, leading to disordered growth, internal stress accumulation, and the formation of microcracks. The optimum pH of 10.8 provides a balanced supersaturation level that enables adequate nucleation density while maintaining controlled crystal growth kinetics, thereby yielding a dense, well-intergrown, and defect-minimized coating. In contrast, the pure LDH coating has a corrosion current density of 7.385 × 10^−6^ A/cm^2^, which is significantly higher than that of the GO/LDH coating, indicating that the introduction of GO helps enhance the protective performance of the coating. The GO/LDH coatings intercalated with different anionic inhibitors all exhibit low corrosion current densities, as shown in Table 1 and Table S3. Among them, the GO/LDH–molybdate coating shows the smallest corrosion current density of 5.126 × 10^−8^ A/cm^2^, followed by the GO/LDH–vanadate coating (1.537 × 10^−7^ A/cm^2^) and the GO/LDH–tungstate coating (4.042 × 10^−6^ A/cm^2^), all of which are significantly lower than those of the non-intercalated GO/LDH coating (6.819 × 10^−7^ A/cm^2^) and the LDH coating (1.333 × 10^−5^ A/cm^2^). These results indicate that all three anionic corrosion inhibitors effectively improve the corrosion resistance of the coating, with the molybdate ion (MoO_4_^2−^) exhibiting the most significant inhibition effect [36,37]. The pH 10.8 LDHs, pH 10.8 GO/LDHs, and the LDHs and GO/LDHs used in the inhibitor intercalation section were not from the same fabrication batches. Due to inevitable experimental fluctuations arising from different batches of hydrothermal synthesis, substrate pretreatment, and electrochemical testing, the corrosion current densities exhibit variations within a certain range. However, these variations in corrosion current density are within one order of magnitude (as shown in Tables S2 and S3), which falls within the range of normal experimental error.

**Figure 5:**
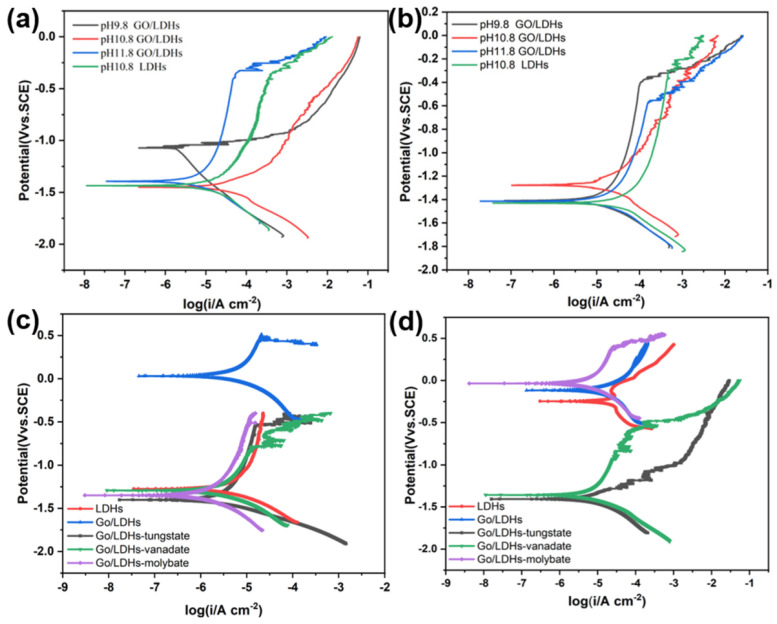
(**a**,**b**) Polarization curves of coatings grown under different pH conditions after soaking in a 3.5 wt.% NaCl solution for 3 days and 6 days; (**c**,**d**) polarization curves of different corrosion inhibitors layers after soaking in a 3.5 wt.% NaCl solution for 3 days and 6 days.

Based on the potentiodynamic polarization curves shown in Figure 5a–d, the corrosion resistance of the coating immersion conditions can be evaluated [37,38]. As shown in Table 2, the pH 10.8 GO/LDH coating exhibits the lowest corrosion current density of 1.93 × 10^−6^ A/cm^2^ after 3 days of immersion, which is significantly lower than that of the pH 9.8 GO/LDH coating (2.84 × 10^−5^ A/cm^2^) and the pH 11.8 GO/LDH coating (8.53 × 10^−6^ A/cm^2^). After 6 days of immersion, the pH 10.8 GO/LDH coating still maintains a relatively low corrosion current density (1.49 × 10^−5^ A/cm^2^), which is lower than those of the pH 9.8 (2.04 × 10^−5^ A/cm^2^) and pH 11.8 (2.18 × 10^−5^ A/cm^2^) coatings. These results indicate that the coating prepared at pH 10.8 exhibits the optimal corrosion resistance. Furthermore, the GO/LDH coatings intercalated with different anionic inhibitors all show low corrosion current densities after 3 and 6 days of immersion [38,39]. Among them, the GO/LDH–molybdate layer exhibits corrosion current densities of 1.384 × 10^−6^ A/cm^2^ and 7.028 × 10^−6^ A/cm^2^ after 3 and 6 days of immersion (Table 3), respectively, both of which are lower than those of the other anionic intercalated coatings, the non-intercalated GO/LDH coating, and the LDH coating. Notably, the GO/LDH–tungstate coating exhibited a higher corrosion current density (4.042 × 10^−6^ A/cm^2^) than the unmodified GO/LDH coating (6.819 × 10^−7^ A/cm^2^) after 30 min of immersion, indicating an initial deterioration in corrosion resistance. This may be attributed to the larger ionic radius of tungstate compared to molybdate and vanadate, which could induce interlayer expansion or local structural distortion in the LDH framework, reducing coating compactness in the short term. Furthermore, the slower passivation kinetics of tungstate may delay the formation of a protective film at the coating/metal interface. However, after 3 and 6 days of immersion, the tungstate-intercalated coating showed lower corrosion current densities than the unmodified GO/LDH coating (Table 3), suggesting that tungstate provides moderate long-term protection once the coating stabilizes. These findings demonstrate that all three anionic corrosion inhibitors effectively enhance the corrosion resistance of the coating [40,41].

### 3.3. Electrochemical Impedance Spectroscopy Analysis

**Figure 6:**
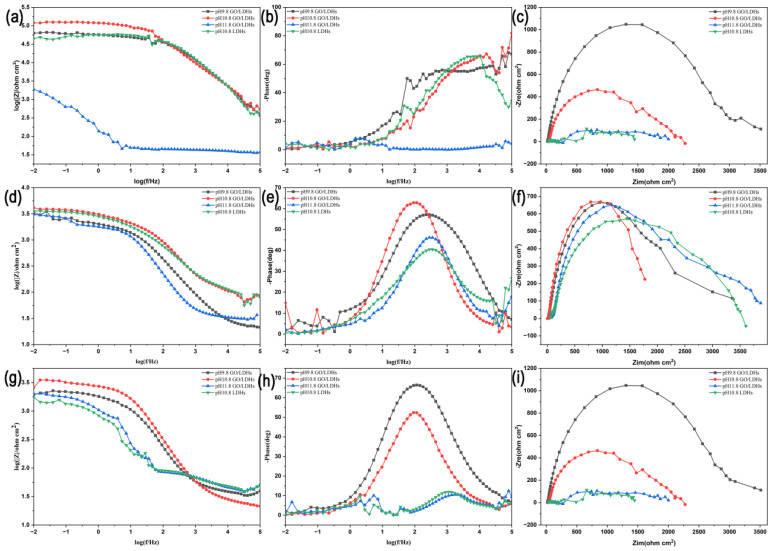
The EIS curves of pH 9.8 GO/LDH coating, pH 10.8 GO/LDH coating, pH 11.8 GO/LDH coating and pH 10.8 LDH coating after (**a**–**c**) 30 min, (**d**–**f**) 3 days and (**g**–**i**) 6 days immersion in the 3.5 wt% NaCl solution.

Electrochemical impedance spectroscopy (*EIS*) was employed to analyze the corrosion behavior of coatings grown under different pH conditions after immersion in a 3.5 wt.% NaCl solution for 30 min, 3 days, and 6 days, as shown in Figure 6. Typically, the low-frequency impedance modulus (|Z|_0.01 Hz_) in the Bode plot exhibits a positive correlation with the protective performance of the coating. As observed in Figure 6, the variation in the impedance modulus during immersion differs significantly among the specimens. The phase angle behavior also serves as an indicator for evaluating the protective performance of the coating. When the coating structure is intact with few defects, the phase angle remains at a high level over a wide frequency range [42]. After 30 min of immersion, the pH 10.8 GO/LDH coating exhibited the highest low-frequency impedance modulus, and its phase angle curve maintained a broad high-angle plateau in the high-frequency region (Figure 6a). In contrast, the phase angles of the coatings prepared at pH 9.8 and pH 11.8, as well as the pH 10.8 LDH coating, were relatively lower (Figure 6b), indicating that the composite coating prepared at pH 10.8 possessed superior compactness and barrier performance at the initial stage. After 3 days of immersion, the low-frequency impedance modulus of all specimens decreased, with the pH 10.8 GO/LDH coating showing the smallest reduction and a relatively stable phase angle plateau (Figure 6c,d). After 6 days of immersion, the pH 10.8 GO/LDH coating still maintained a relatively high low-frequency impedance modulus, and its phase angle remained significantly higher than those of the other specimens (Figure 6e,f), further confirming its superior long-term protective performance.

**Figure 7:**
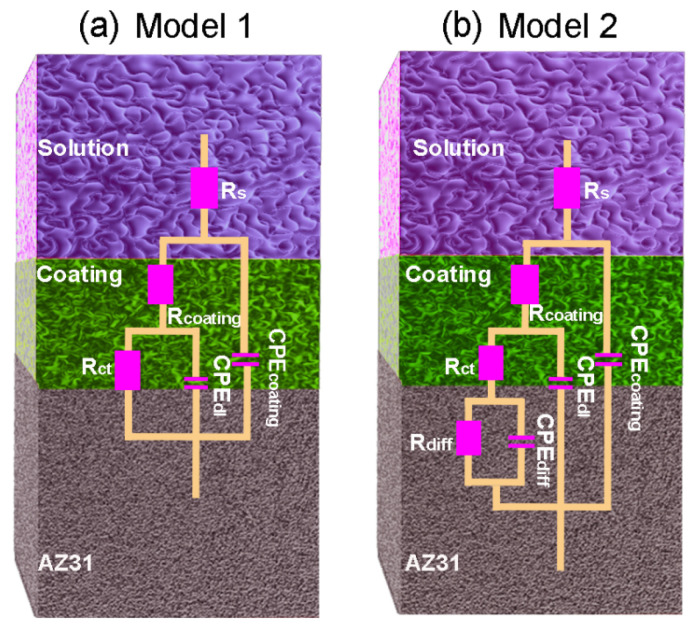
Equivalent circuits used to fit the EIS spectra.

An equivalent electrical circuit (Figure 7) was used to fit the *EIS* data presented in Figure 6, and the obtained electrochemical parameters are listed in Table 4 and Table 5. In the equivalent circuit, *R_coating_* and *R_ct_* represent the coating resistance and charge transfer resistance, respectively, while *CPE_coating_* and *CPE_dl_* correspond to the constant phase elements for coating capacitance and double-layer capacitance, respectively. After 30 min of immersion, the GO/LDH coating prepared at pH 10.8 exhibited the highest *R_coating_* value (6.2 × 10^6^ Ω·cm^2^) and *R_ct_* value (6.8 × 10^6^ Ω·cm^2^), significantly higher than those of the pH 9.8 GO/LDH coating (4.3 × 10^5^ Ω·cm^2^ and 5.1 × 10^4^ Ω·cm^2^), the pH 11.8 GO/LDH coating (7.1 × 10^3^ Ω·cm^2^ and 8.4 × 10^5^ Ω·cm^2^), and the pH 10.8 LDH coating (8.9 × 10^4^ Ω·cm^2^ and 3.8 × 10^5^ Ω·cm^2^), indicating its optimal initial protective performance. As the immersion time extended to 3 days, diffusion-related components, including the constant phase element (*CPE_diff_*) and diffusion resistance (*R_diff_*), were introduced into the equivalent circuits of some specimens, suggesting that the corrosive medium had penetrated into the coating interior. At this stage, the *R_coating_* and *R_ct_* values of the pH 10.8 GO/LDH coating were 6.3 × 10^4^ Ω·cm^2^ and 5.4 × 10^6^ Ω·cm^2^, respectively, still significantly higher than those of the pH 9.8 GO/LDH coating (5.2 × 10^2^ Ω·cm^2^ and 4.1 × 10^4^ Ω·cm^2^), the pH 11.8 GO/LDH coating (4.7 × 10^3^ Ω·cm^2^ and 9.5 × 10^5^ Ω·cm^2^), and the pH 10.8 LDH coating (3.1 × 10^2^ Ω·cm^2^ and 3.3 × 10^4^ Ω·cm^2^). After 6 days of immersion, the *R_coating_* and *R_ct_* values of the pH 10.8 GO/LDH coating were 3.6 × 10^5^ Ω·cm^2^ and 9.2 × 10^4^ Ω·cm^2^, respectively, still considerably higher than those of the other specimens. In contrast, the resistance values of the GO/LDH coatings prepared at pH 9.8 and pH 11.8, as well as the pH 10.8 LDH coating, decreased to varying extents, indicating the progressive degradation of their protective performance. Overall, the GO/LDH composite coating prepared at pH 10.8 maintained relatively stable electrochemical performance throughout the different immersion stages, demonstrating excellent corrosion protection capability.

**Figure 8:**
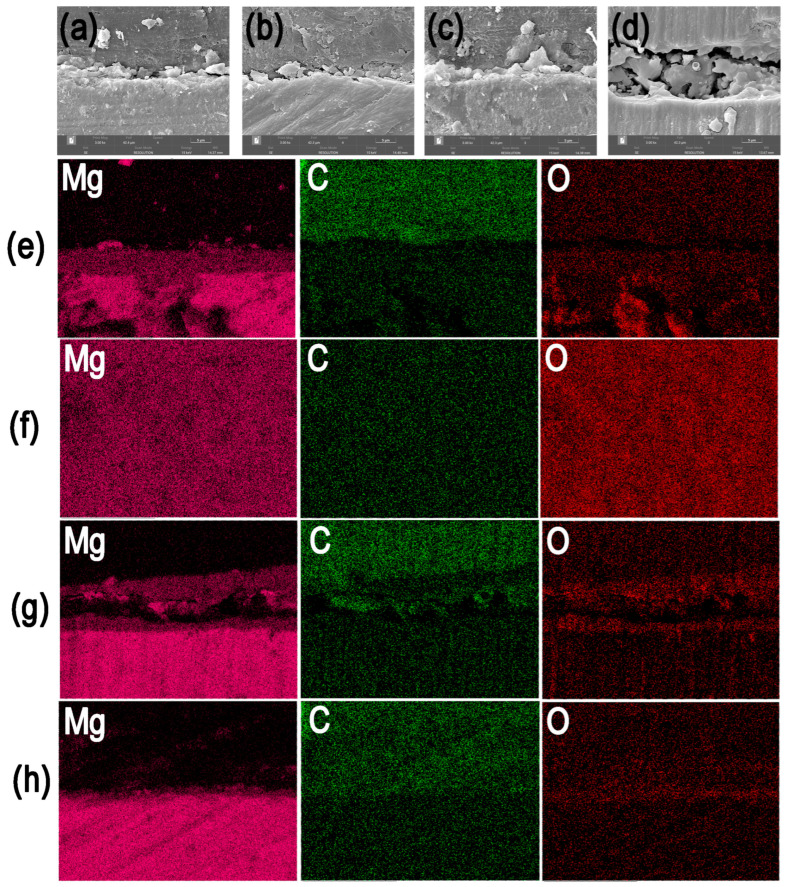
Cross-sectional SEM image after immersion in 3.5 wt% NaCl solution for 6 days: (**a**) pH 9.8 GO/LDHs; (**b**) pH 10.8 GO/LDHs; (**c**) pH 11.8 GO/LDHs; (**d**) pH 10.8 LDHs; EDS line scan of Mg, C, and O elements: (**e**) pH 9.8 GO/LDHs; (**f**) pH 10.8 GO/LDHs; (**g**) pH 11.8 GO/LDHs; (**h**) pH 10.8 LDHs.

To further corroborate the electrochemical evidence with direct structural observations, cross-sectional SEM imaging coupled with EDS elemental line scanning was performed on the coatings after 6 days of immersion in 3.5 wt.% NaCl solution, as shown in Figure 8. The cross-sectional SEM image of the pH 9.8 GO/LDH coating (Figure 8a) reveals pronounced through-thickness cracks and structural discontinuity, indicating severe coating degradation after prolonged immersion. The corresponding EDS line scan (Figure 8e) shows a marked depletion of the C signal (representative of GO) across the coating thickness, suggesting substantial loss of GO from the degraded coating matrix. Similarly, the pH 11.8 GO/LDH coating (Figure 8c) exhibits microcracks and partial delamination at the coating/substrate interface, and its EDS profile (Figure 8g) displays an irregular C distribution with localized carbon-deficient regions, consistent with non-uniform coating deterioration. The pH 10.8 LDH coating without GO (Figure 8d) shows visible porosity and localized thinning, and a correspondingly attenuated signal intensity across the coating (Figure 8h). In striking contrast, the pH 10.8 GO/LDH coating (Figure 8b) retains a dense, compact, and well-adhered cross-sectional morphology with no observable cracks or interfacial separation. Its EDS line scan (Figure 8f) demonstrates a uniform and sustained distribution of C, Mg, and O signals throughout the full coating thickness, confirming the excellent structural integrity and compositional stability of the coating even after 6 days of corrosive exposure. These direct cross-sectional observations provide physical validation of the *EIS* results: the intact morphology and uniform elemental profile of the pH 10.8 GO/LDH coating are fully consistent with its maintained capacitive response and minimal diffusion impedance, whereas the structural degradation observed in the other coatings aligns with the introduction of diffusion components and the pronounced Warburg tails in their Nyquist plots.

**Figure 9:**
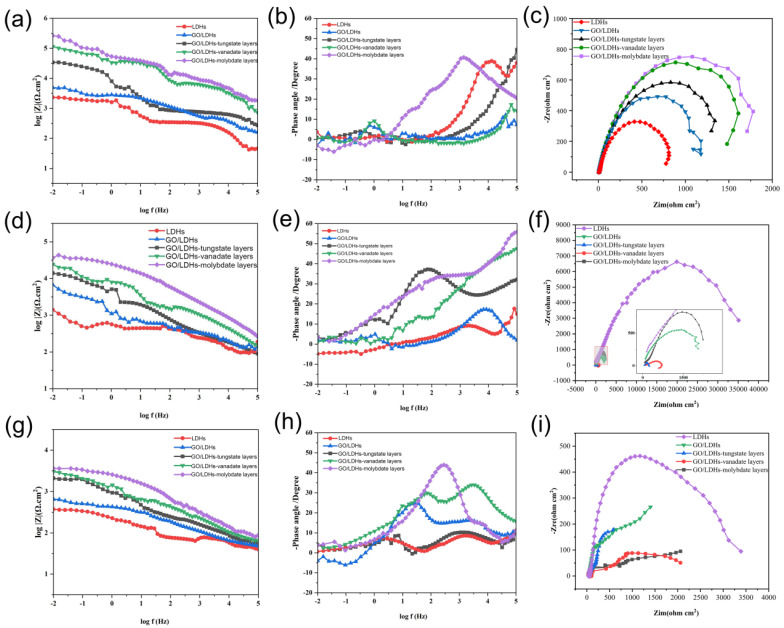
The *EIS* curves of LDHs, GO/LDHs, GO/LDH–tungstate layers, GO/LDH–vanadate layers and GO/LDH–molybdate layers after (**a**–**c**) 30 min, (**d**–**f**) 3 days and (**g**–**i**) 6 days immersion in the 3.5 wt% NaCl solution.

The *EIS* spectra of LDH, GO/LDH, GO/LDH–tungstate, GO/LDH–vanadate, and GO/LDH–molybdate coatings after immersion in a 3.5 wt.% NaCl solution for 30 min, 3 days, and 6 days are presented in Figure 9 and Figure S3. Distinct differences can be observed in the evolution of impedance modulus and phase angle among the various coatings during the immersion process. After 30 min of immersion, the GO/LDH–molybdate coating exhibited the highest low-frequency impedance modulus, and its phase angle curve maintained a broad high-angle plateau in the high-frequency region (Figure 9a,b), whereas the phase angles of the LDH and GO/LDH coatings were relatively lower, indicating that molybdate intercalation significantly enhanced the initial compactness and barrier performance of the coating [43]. An equivalent electrical circuit (Figure 7) was employed to fit the *EIS* data, and the obtained electrochemical parameters are listed in Table 6 and Table 7. In the equivalent circuit, *R_coating_* and *R_ct_* represent the barrier performance and interfacial corrosion resistance of the coating [39], respectively, while *CPE_coating_* and *CPE_dl_* correspond to the constant phase elements for coating capacitance and double-layer capacitance [44,45], respectively. After 30 min of immersion, the GO/LDH–molybdate coating exhibited the highest *R_coating_* value (8.3 × 10^7^ Ω·cm^2^) and *R_ct_* value (4.2 × 10^7^ Ω·cm^2^), significantly higher than those of the unmodified LDH coating and the GO/LDH coating, as well as superior to those of the GO/LDH–tungstate coating and the GO/LDH–vanadate coating, consistent with its highest impedance modulus observed in the nyquist plots (Figure 9c). As the immersion time extended to 3 days, the low-frequency impedance modulus of all coatings decreased to varying extents (Figure 9d). Diffusion-related components, including the constant phase element (*CPE_diff_*) and diffusion resistance (*R_diff_*), were introduced into the equivalent circuits of some coatings, indicating that the corrosive medium had penetrated into the coating interior. At this stage, the GO/LDH–molybdate coating maintained a relatively high impedance modulus and a stable phase angle plateau (Figure 9e,f), with *R_coating_* and *R_ct_* values of 9.2 × 10^4^ Ω·cm^2^ and 1.8 × 10^6^ Ω·cm^2^, respectively, significantly higher than those of the other coatings. Moreover, no diffusion-related components appeared in its equivalent circuit, suggesting that the coating still retained good barrier performance. After 6 days of immersion, the low-frequency impedance modulus of the GO/LDH–molybdate coating remained at a relatively high level (Figure 9g), and its phase angle and nyquist radius of the capacitive arc was significantly higher than those of the other specimens (Figure 9h,i), with *R_coating_* and *R_ct_* values of 7.6 × 10^5^ Ω·cm^2^ and 1.3 × 10^7^ Ω·cm^2^, respectively. In contrast, the impedance modulus of the LDH and GO/LDH coatings decreased markedly, with resistance values falling to the order of 10^2^–10^3^ Ω·cm^2^, and their phase angles in the high-frequency region also decreased significantly, indicating that their protective performance had largely failed. Overall, the GO/LDH–molybdate coating exhibited the optimal electrochemical performance throughout the different immersion stages, demonstrating the best long-term corrosion protection capability.

### 3.4. Corrosion Protection Mechanism

**Figure 10:**
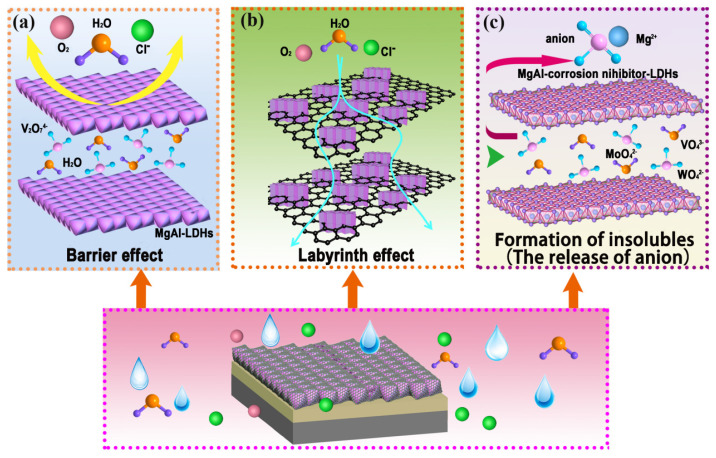
Schematic illustration of the corrosion protection mechanism of the anionic corrosion inhibitor-intercalated GO/LDH composite coating (arrows indicate the corrosion protection mechanism of the species in each subfigure.): (**a**) LDHs block the invasion of corrosive media, (**b**) GO prolongs the diffusion path of corrosive media, (**c**) Release of inhibitor anions forms an insoluble protective layer.

**Figure 11:**
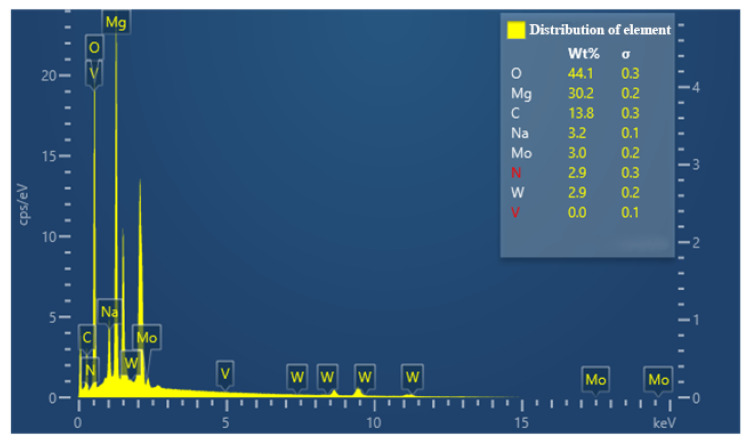
EDS spectrum and surface elemental composition (wt%) of the coating/metal interface after immersion. The results show the presence of O, Mg, C, Mo, W, etc., with a molybdenum content of approximately 3.0 wt%.

Based on the findings of this study, the corrosion protection mechanism of the GO/LDH composite coating can be elucidated from two aspects: physical barrier and chemical inhibition. First, through a pH-regulated substrate-assisted hydrothermal deposition process, a dense and defect-reduced GO/LDH coating was obtained at pH 10.8. In this coating, LDHs adhere tightly to the magnesium alloy substrate via electrostatic interactions or chemical bonding (Figure 10a), providing a stable interface for the subsequent loading of GO. The GO nanosheets, owing to their large specific surface area and abundant oxygen-containing functional groups, firmly bond with the LDH layer, forming a multilayered stacked composite structure. This structure effectively prolongs the diffusion path of corrosive media, thereby significantly enhancing the physical barrier performance of the coating (Figure 10b). Second, after the intercalation of anionic corrosion inhibitors such as molybdate, vanadate, and tungstate into the interlayer spaces of the GO/LDH coating, these inhibitors can be controllably released through an ion exchange process when corrosive media penetrate the coating. Subsequently, they may form a stable passive film at the coating/metal interface, suppressing cathodic reactions and thereby providing active corrosion protection (Figure 10c). Among them, the EDS mapping results indicate the formation of a molybdate-rich surface layer, which may play a protective role (Figure 11). The red labeling of N and V in Figure 11 may be due to peak overlap interference. Figure S4 shows the XPS analysis (Mo 3d spectrum) of the GO/LDH–molybdate coating after immersion corrosion testing. The results show that both Mo(VI) and Mo(IV) species are present on the coating surface after corrosion. Specifically, the peaks located at ~232.5 eV and ~235.6 eV are assigned to Mo(VI) species, confirming that the intercalated molybdate ions are released during the corrosion process and participate in film formation. Meanwhile, the peaks at ~229.3 eV and ~232.4 eV are assigned to Mo(IV) species, indicating a partial reduction of Mo(VI) [46]. These findings provide direct evidence for the formation of a Mo-containing passive film on the metal surface, strongly supporting the proposed passive film mechanism. Direct evidence such as XPS and cross-sectional SEM/TEM is still needed to verify the nature of this layer as a stable passive film. In summary, the GO/LDH–molybdate composite coating achieves efficient and long-term corrosion protection for the magnesium alloy substrate through the synergistic effect of a physical barrier and chemical inhibition.

## 4. Conclusions

In this study, a GO/LDH composite coating was successfully fabricated on the surface of an anodized magnesium alloy via a pH-regulated substrate-assisted hydrothermal deposition method. The effects of pH on the microstructure and corrosion resistance of the coating were systematically investigated, and three anionic corrosion inhibitors, namely molybdate, vanadate, and tungstate, were subsequently intercalated into the coating. The results showed that the GO/LDH coating prepared at pH 10.8 exhibited the densest structure with the fewest defects, demonstrating optimal physical barrier performance. After intercalation with molybdate and vanadate, the corrosion resistance was significantly enhanced. Tungstate intercalation, however, led to an initial deterioration in corrosion resistance, though long-term protection was still improved compared to the unmodified GO/LDH coating. Among them, the molybdate-intercalated GO/LDH coating maintained the highest low-frequency impedance modulus and charge transfer resistance throughout the long-term immersion period, indicating the best corrosion resistance. The protection mechanism of this composite coating can be attributed to the synergistic effect of physical barriers and chemical inhibition. The dense multilayer structure effectively impedes the penetration of corrosive media, while the intercalated anionic corrosion inhibitors are controllably released upon corrosive stimulation, forming a stable passive film at the coating/metal interface. This study provides an effective strategy for the design of high-performance anti-corrosion coatings for magnesium alloy surfaces.

## Figures and Tables

**Table 1:** Polarization curve parameters for coatings under different pH conditions and interlayering of different corrosion inhibitors after 30 min of immersion.

Soak Time	Specimen	*E_corr_* (V_SCE_)	*i_corr_* (A/cm^2^)
30 min 30 min	pH 9.8 GO/LDHs	−1.376 ± 0.021	(6.03 ± 0.52) × 10^−7^
	pH 10.8 GO/LDHs	−1.423 ± 0.018	(3.52 ± 0.31) × 10^−7^
	pH 11.8 GO/LDHs	−1.362 ± 0.025	(5.86 ± 0.48) × 10^−7^
	pH 10.8 LDHs	−1.339 ± 0.030	(7.39 ± 0.67) × 10^−6^
	LDHs	0.2485 ± 0.084	(1.333 ± 0.27) × 10^−6^
	GO/LDHs	−0.801 ± 0.035	(6.82 ± 0.58) × 10^−7^
	GO/LDH–tungstate	−0.968 ± 0.028	(4.04 ± 0.39) × 10^−6^
	GO/LDH–vanadate	−0.514 ± 0.022	(1.54 ± 0.13) × 10^−7^
	GO/LDH–molybdate	−0.994 ± 0.019	(5.13 ± 0.42) × 10^−8^

**Table 2:** Polarization curve parameters of coatings grown under different pH conditions after 3 days and 6 days of immersion.

Soak Time	Specimen	*E_corr_* (V_SCE_)	*i_corr_* (A/cm^2^)
3 days	pH 9.8 GO/LDHs	−1.450 ± 0.028	(2.84 ± 0.31) × 10^−5^
	pH 10.8 GO/LDHs	−1.072 ± 0.022	(1.93 ± 0.18) × 10^−6^
	pH 11.8 GO/LDHs	−1.394 ± 0.026	(8.53 ± 0.79) × 10^−6^
	pH 10.8 LDHs	−1.435 ± 0.031	(1.54 ± 0.16) × 10^−5^
6 days	pH 9.8 GO/LDHs	−1.278 ± 0.024	(2.04 ± 0.22) × 10^−5^
	pH 10.8 GO/LDHs	−1.410 ± 0.020	(1.49 ± 0.14) × 10^−5^
	pH 11.8 GO/LDHs	−1.414 ± 0.027	(2.18 ± 0.23) × 10^−5^
	pH 10.8 LDHs	−1.431 ± 0.029	(3.28 ± 0.35) × 10^−5^

**Table 3:** Polarization curve parameters of coatings of different corrosion inhibitors layers after 3 days and 6 days of immersion.

Soak Time	Specimen	*E_corr_* (V_SCE_)	*i_corr_* (A/cm^2^)
3 days	LDHs	−1.286 ± 0.030	(4.49 ± 0.48) × 10^−6^
	GO/LDHs	0.030 ± 0.035	(5.90 ± 0.62) × 10^−6^
	GO/LDH–tungstate	−1.402 ± 0.025	(2.39 ± 0.25) × 10^−6^
	GO/LDH–vanadate	−1.294 ± 0.028	(2.93 ± 0.31) × 10^−6^
	GO/LDH–molybdate	−1.350 ± 0.022	(1.38 ± 0.14) × 10^−6^
6 days	LDHs	−0.248 ± 0.042	(1.77 ± 0.19) × 10^−5^
	GO/LDHs	−0.117 ± 0.038	(1.85 ± 0.20) × 10^−5^
	GO/LDH–tungstate	−1.406 ± 0.026	(1.01 ± 0.11) × 10^−5^
	GO/LDH–vanadate	−1.358 ± 0.024	(8.08 ± 0.86) × 10^−6^
	GO/LDH–molybdate	−0.035 ± 0.040	(7.03 ± 0.75) × 10^−6^

**Table 4:** The electrochemical parameters were obtained by fitting Figure 6 (first data set).

Immersion Period	Specimens	*CPE_coating_*		*R_coating_* (Ω cm^2^)	*CPE_dl_*		*R_ct_* (Ω cm^2^)
		*Y*_0_ (Ω^−1^ cm^−2^ S^n^)	*n*		*Y*_0_ (Ω^−1^ cm^−2^ S^n^)	*n*	
30 min	pH9.8 GO/LDHs	8.4 × 10^−6^	0.6	4.3 × 10^5^	3.6 × 10^−5^	0.8	5.1 × 10^4^
	pH10.8 GO/LDHs	4.2 × 10^−7^	0.7	6.2 × 10^6^	5.2 × 10^−6^	0.9	6.8 × 10^6^
	pH11.8 GO/LDHs	7.1 × 10^−5^	0.8	7.1 × 10^3^	7.4 × 10^−6^	1.0	8.4 × 10^5^
	pH10.8 LDHs	1.6 × 10^−6^	0.8	8.9 × 10^4^	7.6 × 10^−4^	0.7	3.8 × 10^5^
3 days	pH9.8 GO/LDHs	8.2 × 10^−5^	0.5	5.2 × 10^2^	3.6 × 10^−4^	0.9	4.1 × 10^4^
	pH10.8 GO/LDHs	6.4 × 10^−6^	0.9	6.3 × 10^4^	3.5 × 10^−6^	1.0	5.4 × 10^6^
	pH11.8 GO/LDHs	6.2 × 10^−5^	1.0	4.7 × 10^3^	5.3 × 10^−4^	0.9	9.5 × 10^5^
	pH10.8 LDHs	7.1 × 10^−5^	0.7	3.1 × 10^2^	1.4 × 10^−3^	0.8	3.3 × 10^4^
6 days	pH9.8 GO/LDHs	6.3 × 10^−3^	0.6	7.8 × 10^2^	1.3 × 10^−2^	0.8	4.7 × 10^2^
	pH10.8 GO/LDHs	4.8 × 10^−4^	0.9	3.6 × 10^5^	8.5 × 10^−5^	1.0	9.2 × 10^4^
	pH11.8 GO/LDHs	5.9 × 10^−3^	0.8	4.7 × 10^4^	9.1 × 10^−4^	0.9	6.6 × 10^4^
	pH10.8 LDHs	3.2 × 10^−2^	0.7	9.1 × 10^3^	4.3 × 10^−2^	0.7	7.3 × 10^4^

**Table 5:** The electrochemical parameters were obtained by fitting Figure 6 (second data set).

Immersion Period	Specimens	*CPE_diff_*		*R_diff_* (Ω cm^2^)	*χ* ^2^
		*Y*_0_ (Ω^−1^ cm^−2^ S^n^)	*n*		
30 min	pH9.8 GO/LDHs	−	−	−	2.3 × 10^−3^
	pH10.8 GO/LDHs	−	−	−	5.1 × 10^−3^
	pH11.8 GO/LDHs	−	−	−	3.8 × 10^−3^
	pH10.8 LDHs	−	−	−	4.5 × 10^−3^
3 days	pH9.8 GO/LDHs	3.6 × 10^−2^	0.9	8.6 × 10^2^	2.1 × 10^−3^
	pH10.8 GO/LDHs	4.2 × 10^−3^	0.6	4.0 × 10^4^	1.9 × 10^−3^
	pH11.8 GO/LDHs	8.8 × 10^−3^	0.7	5.9 × 10^3^	3.0 × 10^−3^
	pH10.8 LDHs	3.2 × 10^−3^	0.8	6.5 × 10^3^	9.2 × 10^−3^
6 days	pH9.8 GO/LDHs	5.1 × 10^−2^	0.8	3.9 × 10^2^	2.0 × 10^−3^
	pH10.8 GO/LDHs	3.2 × 10^−2^	0.8	9.2 × 10^3^	8.3 × 10^−3^
	pH11.8 GO/LDHs	3.6 × 10^−2^	0.7	4.1 × 10^2^	1.7 × 10^−3^
	pH10.8 LDHs	7.8 × 10^−4^	0.9	8.6 × 10^2^	5.8 × 10^−3^

**Table 6:** The electrochemical parameters were obtained by fitting Figure 9 (first data set).

Immersion Period	Specimens	*CPE_coating_*		*R_coating_* (Ω cm^2^)	*CPE_dl_*		*R_ct_* (Ω cm^2^)
		*Y*_0_ (Ω^−1^ cm^−2^ S^n^)	*n*		*Y*_0_ (Ω^−1^ cm^−2^ S^n^)	*n*	
30 min	LDHs	8.2 × 10^−5^	0.8	5.8 × 10^4^	7.6 × 10^−5^	0.7	5.1 × 10^4^
	GO/LDHs	5.1 × 10^−5^	0.9	1.9 × 10^5^	4.3 × 10^−6^	0.9	2.2 × 10^5^
	GO/LDH–tungstate	3.6 × 10^−6^	0.6	2.5 × 10^5^	8.9 × 10^−6^	0.6	3.7 × 10^5^
	GO/LDH–vanadate	7.4 × 10^−7^	0.7	6.3 × 10^6^	2.1 × 10^−7^	0.8	8.4 × 10^6^
	GO/LDH–molybdate	9.8 × 10^−9^	0.9	8.3 × 10^7^	6.7 × 10^−7^	0.5	4.2 × 10^7^
3 days	LDHs	4.3 × 10^−4^	0.7	4.5 × 10^2^	9.1 × 10^−4^	0.9	1.3 × 10^4^
	GO/LDHs	7.2 × 10^−4^	0.6	2.5 × 10^3^	5.8 × 10^−5^	0.5	9.2 × 10^3^
	GO/LDH–tungstate	1.9 × 10^−5^	0.9	8.8 × 10^3^	7.3 × 10^−5^	0.7	4.8 × 10^5^
	GO/LDH–vanadate	2.7 × 10^−6^	0.8	1.8 × 10^4^	5.4 × 10^−6^	0.6	2.1 × 10^5^
	GO/LDH–molybdate	6.5 × 10^−7^	0.7	9.2 × 10^4^	1.2 × 10^−6^	0.8	1.8 × 10^6^
6 days	LDHs	3.7 × 10^−3^	0.9	3.4 × 10^2^	6.8 × 10^−2^	0.8	4.5 × 10^2^
	GO/LDHs	5.8 × 10^−4^	0.8	2.6 × 10^2^	2.3 × 10^−3^	0.7	1.8 × 10^2^
	GO/LDH–tungstate	4.2 × 10^−4^	0.6	9.2 × 10^3^	3.7 × 10^−3^	0.9	3.5 × 10^3^
	GO/LDH–vanadate	8.1 × 10^−6^	0.8	3.6 × 10^4^	5.9 × 10^−6^	0.5	2.2 × 10^5^
	GO/LDH–molybdate	2.9 × 10^−7^	0.7	7.6 × 10^5^	1.1 × 10^−7^	0.6	1.3 × 10^7^

**Table 7:** The electrochemical parameters were obtained by fitting Figure 9 (second data set).

Immersion Period	Specimens	*CPE_diff_*		*R_diff_* (Ω cm^2^)	*χ* ^2^
		*Y*_0_ (Ω^−1^ cm^−2^ S^n^)	*n*		
30 min	LDHs	−	−	−	4.2 × 10^−3^
	GO/LDHs	−	−	−	7.8 × 10^−3^
	GO/LDH–tungstate	−	−	−	6.5 × 10^−3^
	GO/LDH–vanadate	−	−	−	2.1 × 10^−3^
	GO/LDH–molybdate	−	−	−	8.9 × 10^−3^
3 days	LDHs	6.8 × 10^−2^	0.6	5.4 × 10^2^	3.5 × 10^−3^
	GO/LDHs	2.3 × 10^−3^	0.9	7.8 × 10^3^	4.3 × 10^−3^
	GO/LDH–tungstate	1.2 × 10^−2^	0.5	2.9 × 10^4^	5.7 × 10^−3^
	GO/LDH–vanadate	8.7 × 10^−3^	0.7	1.3 × 10^3^	1.6 × 10^−3^
	GO/LDH–molybdate	−	−	−	2.4 × 10^−3^
6 days	LDHs	5.5 × 10^−2^	0.5	3.6 × 10^2^	4.8 × 10^−3^
	GO/LDHs	4.1 × 10^−2^	0.7	8.1 × 10^2^	2.9 × 10^−3^
	GO/LDH–tungstate	3.3 × 10^−2^	0.9	4.2 × 10^3^	3.6 × 10^−3^
	GO/LDH–vanadate	2.5 × 10^−3^	0.6	3.7 × 10^4^	5.1 × 10^−3^
	GO/LDH–molybdate	1.1 × 10^−3^	0.8	1.5 × 10^4^	7.3 × 10^−3^

## Data Availability

The original contributions presented in this study are included in the article/Supplementary Materials. Further inquiries can be directed to the corresponding authors.

## Supplementary Materials

The following supporting information can be downloaded at: https://www.mdpi.com/article/10.3390/ma19122525/s1, Table S1: Summary of preparation conditions for all studied specimens. Figure S1: C 1s XPS spectrum of the pH 9.8 GO/LDHs coating. Figure S2: Polarization curves of the coatings. Table S2: Polarization curve parameters for coatings under different pH conditions after 30 min of immersion. Table S3: Polarization curve parameters for coatings under interlayering of different corrosion inhibitors after 30 min of immersion. Figure S3: EIS spectra of the coatings. Figure S4: Mo 3d XPS spectrum of the GO/LDHs-molybdate coating after immersion.
