# Supplementary material for: Synergistic Enhancement of Corrosion Resistance of GO/LDH Coating on Anodized Magnesium Alloy Surfaces via pH-Regulated In Situ Growth and Anionic Corrosion Inhibitor Intercalation
Source: Materials (Basel). 2026 Jun 11;19(12):2525. doi: 10.3390/ma19122525 (PMC13304309; doi:10.3390/ma19122525)
Supplement: Supplementary file 1 [file materials-19-02525-s001.zip › materials-4251220-supplementary.pdf]

Supplementry Material

Synergistic Enhancement of Corrosion Resistance of GO/LDH Coating on Anodized Magnesium Alloy Surfaces via pH-Regulated In Situ Growth and Anionic Corrosion Inhibitor Intercalation

Yanning Chen [1,*], Tongqing Wang [1], Manyu Liu [1], Hao Ji [1], Yuehua Sun [1], Zhen Sun [2], Chengsi Zheng [1], Zhenya Zhang [1] and Mingya Zhang [1,*]

[1] Key Laboratory of Green Fabrication and Surface Technology of Advanced Metal Materials, Ministry of Education, School of Materials Science and Engineering, Anhui University of Technology, Maanshan 243002, China
[2] Inner Mongolia Key Laboratory of New Materials and Surface Engineering, School of Materials Science and Engineering, Mongolia University of Technology, Hohhot 010051, China
* Correspondence: ynchen@ahut.edu.cn (Y.C.); ahutzmh@163.com (M.Z.)

Content

Table S1: Summary of preparation conditions

Figure S1: C 1s XPS spectrum

Figure S2: Polarization curves of the coatings

Figure S3: EIS spectra of the coatings

Table S2 & S3: Additional polarization parameters (for reference)

Figure S4: Mo 3d XPS spectrum

Table S1 Summary of preparation conditions for all studied specimens

Specimen name	Substrate pretreatment	Coating preparation process	Key process parameters
LDHs	Grinding, polishing, anodizing	One-step hydrothermal growth	pH 10.8, 125 °C, 12 h
pH 9.8 GO/LDHs	Grinding, polishing, anodizing	One-step hydrothermal growth with GO addition	pH 9.8, 125 °C, 12 h
pH 10.8 GO/LDHs	Grinding, polishing, anodizing	One-step hydrothermal growth with GO addition	pH 10.8, 125 °C, 12 h
pH 11.8 GO/LDHs	Grinding, polishing, anodizing	One-step hydrothermal growth with GO addition	pH 11.8, 125 °C, 12 h
GO/LDHs-vanadate	Grinding, polishing, anodizing	Hydrothermal growth (pH 10.8) + secondary hydrothermal intercalation	60 °C, 6 h, 0.1 M $Na_3VO_4 \cdot 12H_2O$
GO/LDHs-molybdate	Grinding, polishing, anodizing	Hydrothermal growth (pH 10.8) + secondary hydrothermal intercalation	60 °C, 6 h, 0.1 M $Na_2MoO_4 \cdot 12H_2O$
GO/LDHs-tungstate	Grinding, polishing, anodizing	Hydrothermal growth (pH 10.8) + secondary hydrothermal intercalation	60 °C, 6 h, 0.1 M $Na_2WO_4 \cdot 12H_2O$

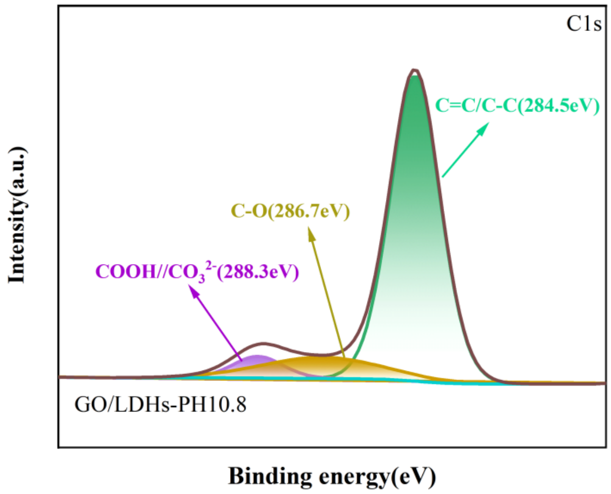

Figure S1. C 1s XPS spectrum of the pH 9.8 GO/LDHs coating.

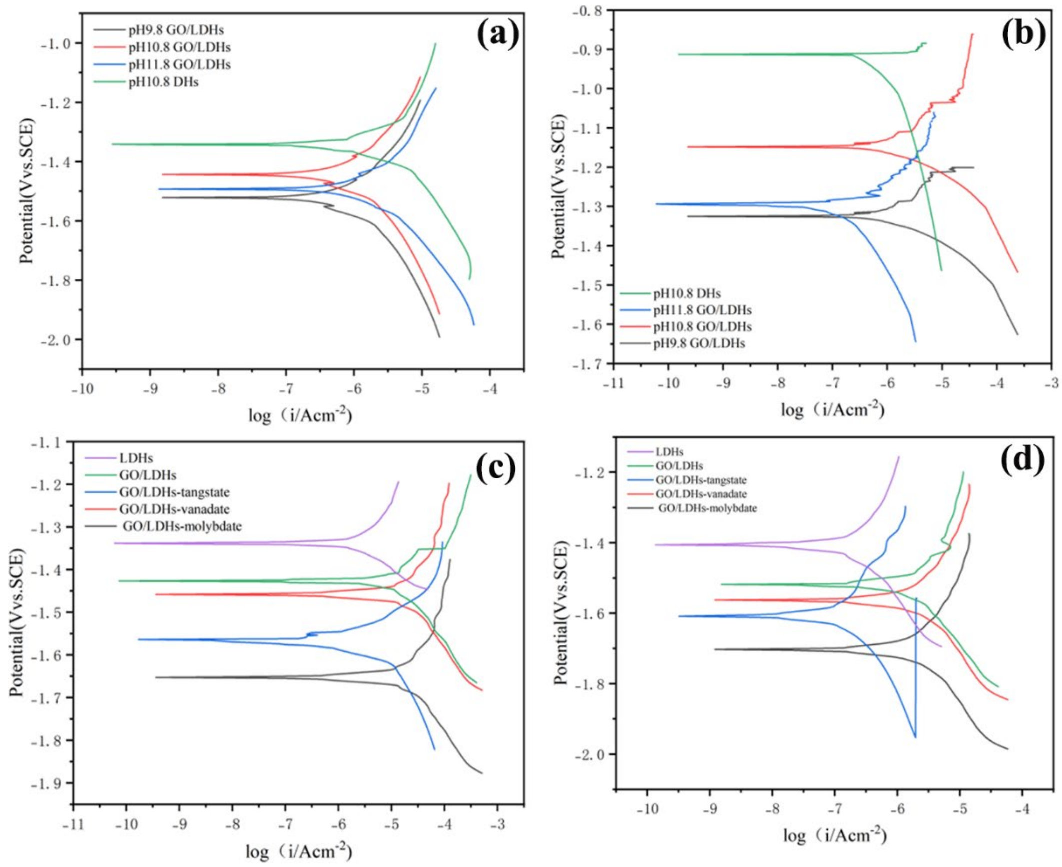

Figure S2. Polarization curves of the coatings.

Table S2. Polarization curve parameters for coatings under different pH conditions after 30 minutes of immersion

soak time	specimen	E_{corr}（V_{SCE}）	i_{corr} (A/cm²)
30min	pH 9.8 GO/LDHs	-1.5178	1.508×10^{-6}
	pH10.8 GO/LDHs	-1.4736	2.111×10^{-7}
	pH11.8 GO/LDHs	-1.4975	1.054×10^{-6}
	pH10.8 LDHs	-1.3569	3.323×10^{-6}
30min	pH 9.8 GO/LDHs	-0.9866	4.265×10^{-6}
	pH10.8 GO/LDHs	-1.2841	5.455×10^{-7}
	pH11.8 GO/LDHs	-1.2532	6.063×10^{-7}
	pH10.8 LDHs	-1.3250	3.381×10^{-6}

Table S3. Polarization curve parameters for coatings under interlayering of different corrosion inhibitors after 30 minutes of immersion

soak time	specimen	E_{corr} (V_{SCE})	i_{corr} (A/cm²)
30min	LDHs	-1.3451	1.052×10^{-6}
	GO/LDHs	-1.4229	8.274×10^{-7}
	GO/LDHs-tungstate	-1.5681	3.856×10^{-6}
	GO/LDHs-vanadate	-1.4672	2.113×10^{-7}
	GO/LDHs-molybdate	-1.6632	7.894×10^{-8}
30min	LDHs	-1.4121	1.768×10^{-6}
	GO/LDHs	-1.5233	5.432×10^{-7}
	GO/LDHs-tungstate	-1.6160	4.991×10^{-6}
	GO/LDHs-vanadate	-1.5275	9.567×10^{-8}
	GO/LDHs-molybdate	-1.7099	3.205×10^{-8}

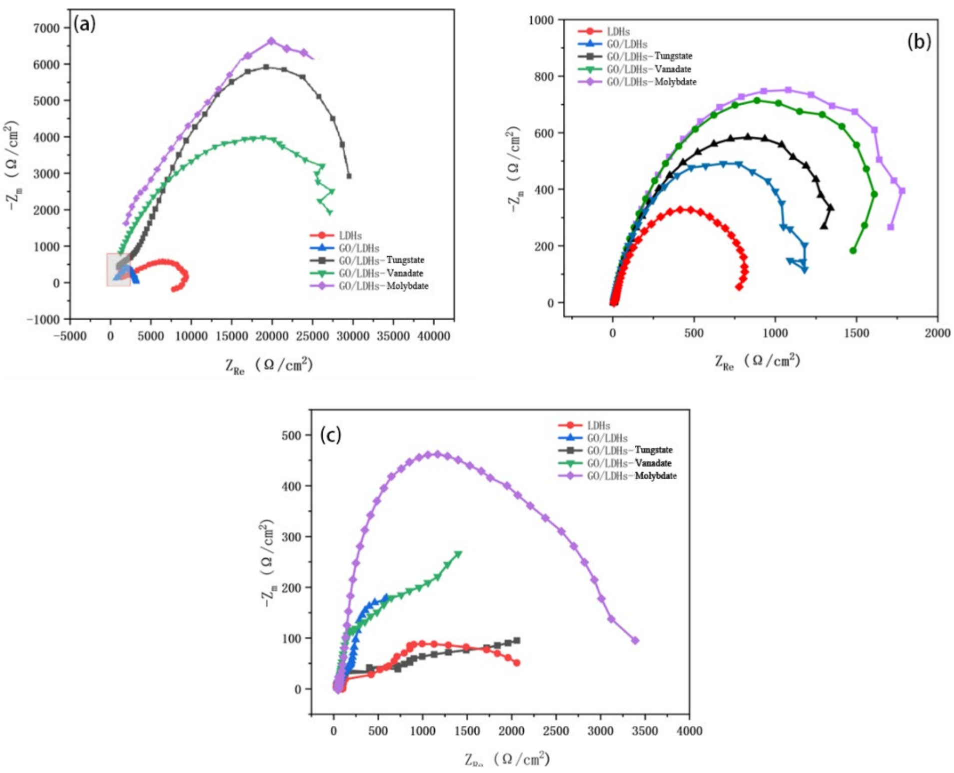

Figure S3. EIS spectra of the coatings.

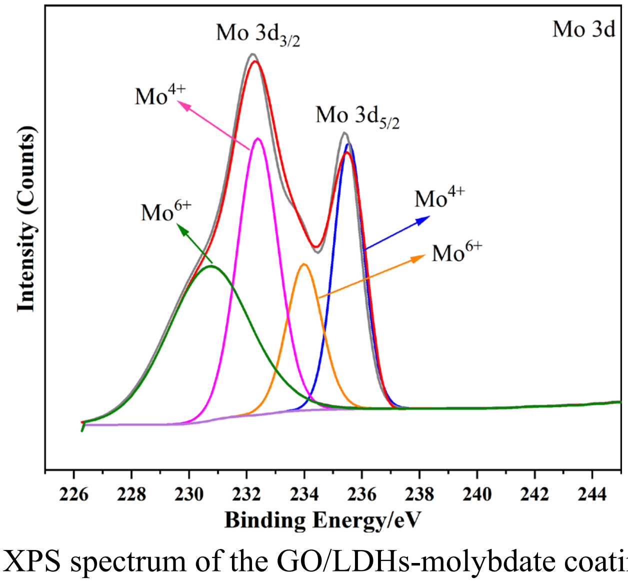

Figure S4. Mo 3d XPS spectrum of the GO/LDHs-molybdate coating after immersion
